# Online Mindfulness Intervention for Inflammatory Bowel Disease: Adherence and Efficacy

**DOI:** 10.3389/fpsyg.2021.709899

**Published:** 2022-03-24

**Authors:** Leila Forbes, Susan K. Johnson

**Affiliations:** ^1^BASE Cognitive Behavioral, Charlotte, NC, United States; ^2^Department of Psychological Science, University of North Carolina at Charlotte, Charlotte, NC, United States

**Keywords:** mindfulness, online intervention, Inflammatory Bowel Disease, ulcerative colitis, Crohn’s disease, meditation

## Abstract

The impact of stress and other psychological variables on Inflammatory Bowel Disease (IBD) prognosis, treatment response, and functional level is well-established; however, typical IBD treatment focuses on the physiological pathology of the disease and neglects complementary stress-reducing interventions. Recent pilot studies report the benefits of mindfulness-based interventions (MBIs) in people living with IBD, but are limited by small sample sizes. Recruitment challenges to in-person studies may be in part due to the difficulty IBD patients often have adhering to fixed schedules and travel as a result of IBD symptoms such as pain, fatigue, and incontinence. The current study aimed to address this barrier by offering participants access to online mindfulness training, allowing individuals to engage with intervention materials to fit their own schedule. Online mindfulness programs have gained popularity in recent years, as they increase access and flexibility and decrease cost to the user; however, the dropout rate tends to be high. The current study compared the rate of adherence and efficacy of mindfulness training as a function of level of support: self-guided versus supported. Analysis revealed no significant difference in the benefits received between participants in the two groups; however, a significant difference group (χ^2^ = 15.75; *p* = 0.000, *r* = 0.38) was found in terms of rate of completion, with 44.1% of the supportive group completing the protocol compared to 11.7% of the self-guided. Common challenges to meditation were measured, but did not significantly predict adherence to the intervention, and experience of these challenges did not significantly change (increase or decrease) over the duration of the study. Implications of the current research, future directions for the use of MBI for IBD patients, and a discussion of methodological considerations are provided.

## Introduction

### Inflammatory Bowel Disease

Inflammatory Bowel Disease (IBD) refers to chronic immune system disorders affecting the digestive tract (Shih and Targan, 2009; McCombie et al., 2013). The complex pathogenesis is believed to involve genetic, environmental, and immunological factors (Bouma and Strober, 2003; Gearry et al., 2010). The prevalence of the most common forms of IBD, Crohn’s disease (CD), and ulcerative colitis (UC), is accelerating in developing countries, and remains high in western countries at over 0.3%, and higher still in countries such as the United States at 1.3% (Ng et al., 2018).

Inflammatory Bowel Disease can cause severe abdominal pain and gastrointestinal symptoms. The disease is typically progressive, often requires surgery, and increases the risk of colon cancer (Nguyen et al., 2009). Pharmacological treatment is the primary intervention (Bernal et al., 2006; Jackson et al., 2010); however, medication adherence is often low, and even when compliant, a third of IBD patients continue to experience symptoms affecting their functioning.

In addition to the physical toll, IBD also impacts psychological health (Graff et al., 2009; McCombie et al., 2013). In a study of 52,782 patients with IBD, Szigethy et al. (2021) found that 43% had a mental health diagnosis. The most common diagnoses are depression and anxiety, which remain high even when patients are in remission (Mittermaier et al., 2004; Graff et al., 2009), and as compared to other chronic illness populations (Walker et al., 2008; Hauser et al., 2011). A recent review suggests that alexithymia tendencies in IBD that may lead to impaired emotional recognition over time (Martino et al., 2020). Due to the chronic nature of IBD, patients often find themselves in a persistent state of psychological distress, anticipating and coping with symptoms (Rubio et al., 2016).

### Mindfulness-Based Interventions

Numerous studies have examined the effect of mindfulness-based interventions (MBIs) on chronic illness (Carson et al., 2017; Scott-Sheldon et al., 2019; Simpson et al., 2020). Mindfulness training may have particular benefits for chronic inflammatory conditions, such as IBD, through stress buffering mechanisms (Rosenkranz et al., 2013; Zhou et al., 2020). Mindfulness training aims to alter both threat appraisal and utilization of coping skills. In a review of MBIs for various chronic illnesses, Greeson and Chin (2019) found that stress reduction was the most robust benefit, while effects on objective measures of disease generally remain non-significant. Ewais et al. (2019) conducted a meta-analysis of MBIs and yoga for IBD. They found eight randomized controlled trials of sufficient quality indicating that MBIs were effective in reducing stress, depression, and improving quality of life. Effects on anxiety and physical outcomes were equivocal and not statistically significant. Although generally positive results were reported in Ewais et al.’s (2019) meta-analysis, several studies have found no benefit of MBIs in IBD. For example, Berrill et al. (2014) found no significant effect of a Mindfulness-Based Stress Reduction (MBSR) program on stress level, quality of life, or disease remission in IBD patients. Berrill et al. (2014) also noted a high drop-out rate wherein 24% of participants failed to attend a single appointment and the majority of the sample was lost to follow-up. This was one of few studies which included the rate of adherence to MBI protocols.

### Online Interventions

Scheduled, face-to-face mindfulness classes may be daunting for people with IBD, who may experience fatigue, sudden onset of symptoms, as well as potential exposure to novel viruses such as COVID-19. Delivering interventions online may provide more people with IBD benefits from mindfulness training by allowing for in home practice and scheduling flexibility. Under the category of “self-help,” researchers have begun to examine the efficacy of MBIs delivered through on-line programs and phone apps (Lewis et al., 2012; Chadi et al., 2020).

Despite the ongoing medical and psychological factors involved in IBD, patients spend an average of only 3 h per year obtaining medical care (Keefer and Kane, 2016). Thus, IBD patients engage heavily in self-management of the disease. To date, only one study utilized an online format to deliver an 8-week cognitive behavioral therapy (CBT) intervention specifically to individuals with IBD (McCombie et al., 2016). The intervention was effective in increasing the use of healthy coping techniques and quality of life, but did not have an effect on physical symptoms. Only 26% in the CBT group completed all sessions, and improvements were not maintained at 6 months follow-up.

Several studies have assessed the efficacy of online MBIs for other chronic health conditions (Ljótsson et al., 2010; Davis and Zautra, 2013). Specifically, because in chronic pain conditions psychological factors play an important role (Turk and Okifuji, 2002; Varallo et al., 2021). Davis and Zautra (2013) compared participants with fibromyalgia randomized to an online Mindful Socio-Emotional Regulation (MSER) intervention to participants who received online health tips. Results suggested that while daily pain remained unchanged, the participants in the mindfulness group experienced improvements in positive affect, stress management, and engagement in social activities compared to the health tips group (Davis and Zautra, 2013). Notably, only 49% of participants in the MSER and 63% of participants assigned to the health tips group completed all 12 modules. A study of Swedish patients with Irritable Bowel Syndrome (IBS) investigated the benefits of a 10-week, online CBT intervention that included a mindfulness component (Ljótsson et al., 2010). The intervention group demonstrated a significant decrease in IBS symptoms and GI-specific anxiety, as well as improved quality of life compared to a wait-list control. Ljótsson et al. (2011) also conducted a follow-up study demonstrating maintenance of reductions in IBS symptoms and increased quality of life.

Several studies have used smartphone apps to deliver MBIs to chronic illness groups. A qualitative interview study examined feasibility of a customized mindfulness app for women with chronic pelvic pain. Initial enthusiasm for the app was not reflected in actual usage with only 36% using the Mindfulness Meditation (MM) and 42% using a muscle relaxation app at least once. Reasons for low adherence were lack of time, lack of perceived benefit, and technology issues (Ball et al., 2020). Mikolasek et al. (2021) employed a mindfulness and relaxation app for cancer patients who reported improvements in fatigue, sleep, quality of life with reduced distress and anxiety. More distressed patients benefited the most, although only 25% of the sample used the app regularly. In an observational study of the same sample, Mikolasek et al. (2018) found female gender, higher openness to experience, and more depressive symptoms predicted greater adherence to the app intervention. Järvelä-Reijonen et al. (2018) compared Acceptance and Commitment Therapy (ACT) delivered in a face-to-face group sessions to mobile app delivery. Both groups displayed similar levels of improvement on eating behavior.

Spijkerman et al. (2016) reviewed online MBIs for mental health and found 15 randomized controlled trials. Online MBIs were found to have small but significant impacts on depression, anxiety, well-being, and mindfulness. They found the greatest effect for reducing stress. Examining the feasibility of delivering online MBIs for anxiety, Boettcher et al. (2014) found that participants who completed the MBI showed a significantly greater decrease in anxiety, depression, and insomnia than participants in the control group. Querstret et al. (2018) assessed an online MBI in a general population sample and found completers reported lower stress, depression, and anxiety compared to wait list controls. El Morr et al. (2020) found similar results in university students with a web-based mindfulness CBT course. Depression and anxiety were reduced after 8 weeks compared to a wait list group, although perceived stress was not different between groups.

A few studies have examined attrition in online MBIs. Cavanagh and colleagues administered a 14-day online MBI to college students (Cavanagh et al., 2014). Results suggest participants experienced a significant increase in mindfulness, and a significant decrease in stress and anxiety. This study had a high rate of attrition, with only 43% of the intervention group completing the program; however, the majority (61%) of those who completed the intervention indicated that they intended to continue to practice meditation. Howells et al. (2014) compared participants’ scores on measures of well-being following a 10-day MM smartphone app compared to a list making app control. Results indicated that users of the MM app experienced a significant increase in positive affect, and a reduction in depressive symptoms. However, this study experienced a high dropout rate of 77%. A higher level of negative emotion was the only measured characteristic that predicted dropout from the study. A study from our lab (Forbes et al., 2018) administered a 10-session mindfulness training program to 169 college students, naïve to meditation. There was a 47% drop out rate, with lower conscientiousness and trait mindfulness predicting dropout. The participants who completed the program showed increased mindfulness and psychological flexibility compared to baseline.

Online delivery of MBIs have not been studied using IBD patients, and the dropout rates reported in online MBI studies applied to other populations are quite high (Davis and Zautra, 2013; Cavanagh et al., 2014; Howells et al., 2014; Forbes et al., 2018; Ball et al., 2020). Typically, online mindfulness programs are delivered using a self-help model, with minimal support and guidance from research staff. An under-researched area is the impact of support on adherence to online interventions.

### Support in Online Interventions

Increasing the level of support may improve the rate of adherence and extend the efficacy of MBIs. Open-source mindfulness websites with guided meditations, exercises, and educational material abound; however, for the average person, the process of accessing high quality materials, and regularly practicing the skills may be daunting. Access to online MBIs that include email, text messaging, or phone support may improve the adherence and efficacy; however, this question has not been empirically examined.

In terms of online supported MBIs applied to chronic illness populations, Russell et al. (2018) found 10 randomized controlled trials for chronic conditions that compared self-guided to facilitator guided approaches. Online MBIs were more effective than wait list or treatment as usual, yet not more effective than active control conditions. Attrition rates post intervention ranged from 11 to 62%, but attrition was measured so variously that implications for guided approaches were unclear.

### Current Study

The primary research objective of the current study was an examination of the effects of online mindfulness training for patients with IBD. Based on the previous literature, we hypothesized that participants would experience psychological benefits, increase mindfulness (including mindful eating), and may experience enhanced quality of life. The study will also establish the rate of completion of an online mindfulness program for people with IBD, and examine whether the rate of completion or benefits differ depending on the level of support. We hypothesized that participants receiving support would complete the protocol at a higher rate than self-guided participants. In light of the typically high attrition rate in online mindfulness studies, and because the current study teaches mindfulness to naïve meditators, the obstacles experienced while meditating were also examined. Analyses were then conducted to determine whether common challenges to meditation affected the rate of adherence to the intervention.

## Materials and Methods

### Study Design

Members of online IBD support groups were recruited. Participants were at least 18 years old, had received a diagnosis of CD or UC, were interested in learning but had no prior experience with meditation. The recruitment script described the project as a web-based study, examining the efficacy of mindfulness training on people with IBD. It explained that participants would be asked to access guided meditation audio files and complete a number of self-report questionnaires. The time commitment was approximately 2 h per week, a total of 8 h of mindfulness training and 1 h of questionnaires. The recruitment post contained a link to the informed consent. Those who consented were directed to the online study platform.

**FIGURE 1:**
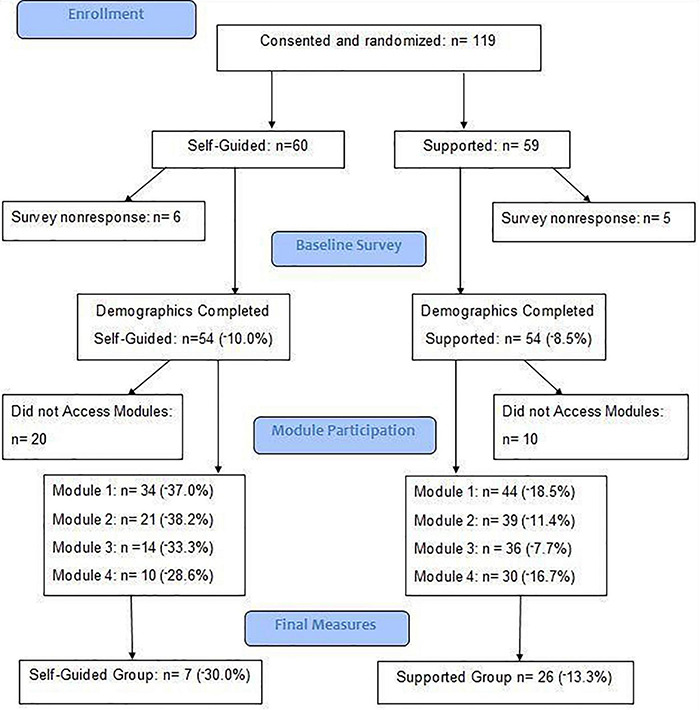
Attrition flow chart.

At the end of data collection, 119 unique cases were logged on Qualtrics (Provo, UT, United States). Of those cases, 11 were excluded from analysis because they opted to “answer questionnaires later” and did not return. One hundred and eight individuals completed at least one baseline measure. Ninety-six participants finished all baseline measures. Seventy-one participants attempted the first meditation and completed the Obstacles Checklist (OBS) and Repetitive Thought Questionnaire. Thirty-three participants accessed all modules, completed the final measures and were included in all levels of analysis. A flow chart of participant data, including attrition rates at all stages, is represented in Figure 1.

The mean age of the sample was 39.27 (SD = 11.46; range = 18–65) and was composed mostly of women (73.1%). The sample was predominantly white (83.3%) with Black participants making up 6.5%, Asian participants 5.6%, Latino participants 3.7%, and 0.9% of the sample identifying as “other.” Half of the participants were married or in a domestic partnership, while 26.9% were single, 17.6% divorced, 2.8% separated, and 2.8% widowed. Data regarding age of diagnosis and disease classification are reported in Table 1. No significant differences in demographic or disease-related variables between participants in the Self-Guided and Supported groups were observed.

**TABLE 1:** Disease characteristics of sample (*n* = 108).

Characteristic	Frequency	Percentage (%)
**Age at diagnosis**		
Under 18 years old	24	22.2
18–24 years old	29	26.9
25–34 years old	35	32.4
35–44 years old	9	8.3
45–54 years old	8	7.4
55+ years old	3	2.8
**IBD classification**		
Mild Crohn’s disease	9	8.3
Moderate Crohn’s disease	31	28.7
Severe Crohn’s disease	11	10.2
Mild ulcerative colitis	17	15.7
Moderate ulcerative colitis	26	24.1
Severe ulcerative colitis	29	13.0

### Procedure

Following online informed consent, participants were randomized to either the supported or self-guided group, then routed to the baseline measures. After the baseline measures were completed, participants continued to a webpage containing a brief introduction to mindfulness and instructions regarding utilizing the study site.

#### Intervention

The intervention was divided into four modules each containing three parts: an introduction, pdf lesson, and four guided meditations related to that particular mindfulness lesson to be accessed on separate occasions. Participants completed modules in succession, and at their own pace, but were instructed to attempt to complete one module per week. The link became inactive after 14 days of inactivity to ensure regular practice. The four modules of the intervention were developed to address psychosocial and behavioral factors relevant to living with IBD. Each module centered around a theme specifically tailored to learning basic principles of mindfulness and addressing psychosocial factors common to IBD. The UCLA Mindful Awareness Research Center guided meditations and pdf lessons used in the intervention were open source.

##### Module 1: Mindfulness of Breathing

Module 1 included an introduction that explained the concept of mindful breathing and mindful awareness. The module included a 5-min “Breathing Meditation” (accessed four times). A pdf titled “Meditation: It’s not what you think” by Kabat-Zinn (2015) was also delivered to participants. These materials provided a foundational understanding and experience of basic meditation.

##### Module 2: Body Scan/Mindful Eating

This module included an introduction to mindful awareness of the body and mindful eating. A 12-min “Breath, Sound, Body” guided meditation was accessed in Module 2, as was a pdf titled, “Mouthfuls of Mindfulness” (Bays, 2012). The Module 2 materials were selected as people with IBD tend to have a complicated relationship with food, and disordered eating is higher in IBD compared to the general population (Defilippis et al., 2015; Ilzarbe et al., 2017). The “Breath, Sound, Body” meditation encourages interacting with bodily sensations, particularly unpleasant sensations with non-judgmental acceptance.

##### Module 3: Meditation for Difficult Situations

This module included an introduction outlining the use of mindfulness in reappraising or coping with difficulties, both physically and emotionally. The module included access to the 7-min “Working with Difficulties” guided meditation and an essay focused on noticing unpleasant thoughts and sensations with curiosity and acceptance.

##### Module 4: Self-Compassion

The final module of the intervention focused on self-compassion. The Module 4 page included an introduction briefly explaining the concept and how it may benefit people with IBD. Research suggests people with IBD often experience guilt and shame stemming from their limitations, symptoms, and reliance on caregivers (Kiebles et al., 2010). Module 4 included a 9-min “Loving Kindness Meditation” and an article titled, “Self-Compassion: The Secret to Empowered Action is Learning Not to Beat Yourself Up” (Seppala, 2011).

#### Supported and Self-Guided Groups

Qualtrics provided an email list of participants randomized to the supported group. Study staff sent participants in the supported group a welcome email that encouraged them to contact study staff with questions or concerns, and to expect reminder emails approximately once a week. Reminder emails were generated when they reached the end of modules 1–3, and encouraged participants to access the next module. A record of participant contact was logged. Participants in the self-guided group navigated through the same modules, but did not receive email support or reminders.

### Measures

#### Demographics

Demographic Questionnaire: participants provided information about their age, gender, ethnicity, educational background, income, marital status, date of IBD diagnosis, and IBD severity classification. This measure was only administered at baseline.

The following measures were administered at baseline and after study completion:

#### Quality of Life

The IBD quality of life (IBDQ) questionnaire (32-items) measures disease-specific quality of life in four domains: bowel symptoms, emotional health, systemic systems, and social function (Irvine, 1993). Responses to items are scored on a 7-point scale in which 1 indicates worst functioning and 7 the best. The total IBDQ points range from 32 to 224, with higher scores reflecting better quality of life. The IBDQ has been shown to be valid and reliable in a clinical setting and sensitive to change during a period of time (Irvine et al., 1994). The Cronbach’s alpha for the current sample was calculated to be 0.93 at baseline administration.

#### Anxiety

The Beck Anxiety Inventory (BAI) is a 21-item, self-administered measure of anxiety that focuses on somatic and cognitive symptoms of anxiety, including nervousness, dizziness, and inability to relax (Beck et al., 1988). Responses are rated on a 4-point Likert scale ranging from 0 (not at all) to 3 (severely). Construct validity studies show good convergence of the BAI with other measures of anxiety including the Hamilton Anxiety Rating Scale, the State Anxiety Inventory, and the anxiety scale of the Symptom Checklist-R (Beck and Steer, 1991). Internal consistency is high in a range of clinical and non-clinical populations (Osman et al., 1993; Creamer et al., 1995). In the current study, Cronbach’s alpha was calculated to be 0.92 at baseline.

#### Stress

Perceived Stress Scale (PSS): a widely used 10-item self-report instrument for measuring the perception of psychological stress (Cohen et al., 1983). The PSS items evaluate the degree to which individuals believe their life has been unpredictable, uncontrollable, and overloaded during the previous month. The PSS-10 has high test-retest reliability (Lee, 2012). Cronbach’s alpha for the current sample was 0.83 at baseline.

#### Depression

The Patient Health Questionnaire (PHQ-9) is a 9-item depression scale used primarily for diagnosing and monitoring treatment of primary care patients (Kroenke et al., 2001). The PHQ-9 allows the user to rate themselves on each of the 9 DSM-IV depression criteria as “0” (not at all) to “3” (nearly every day). The PHQ-9 has demonstrated high internal validity as well as high test-retest reliability. The Cronbach’s alpha for the current sample was 0.89 at baseline.

#### Mindfulness

The Mindful Attention Awareness Scale (MAAS; Brown and Ryan, 2003) is a 15-item, single factor scale measuring the general tendency to be attentive to and aware of the present moment in day-to-day experiences. Using a 6-point Likert scale, participants designate their frequency of particular experiences such as, “I find myself doing things without paying attention.” The MAAS has demonstrated high internal consistency, convergent validity, and discriminant validity in various populations (Brown and Ryan, 2003). The MAAS has also demonstrated sensitivity to change in mindfulness following a brief online mindfulness intervention (Forbes et al., 2018). The Cronbach’s alpha for the current sample was calculated to be 0.88 at baseline administration.

#### Mindful Eating

The Mindful Eating Scale (MES) (Hulbert-Williams et al., 2014) is a 28-item instrument designed to assess the concept of mindfulness as applied to eating behaviors. The scale is self-report with response options on a 4-point Likert scale from (1) never to (4) usually. A number of items are reverse-scored, and subscales measure acceptance, awareness, non-reactivity, routine, and act with awareness. The instrument is considered to be consistent with widely accepted operational definitions of mindfulness. The Cronbach’s alpha in the current sample was calculated to be 0.86 at baseline administration.

The following measures were administered immediately following the first and final mediations:

#### Obstacles

The OBS is a list containing 12 commonly experienced challenges to meditation, including items such as: “becoming too distracted to finish the meditation” and “feeling too anxious/agitated to do the meditation” (Forbes et al., 2018). The participant was asked to rate each item from 1 (“not a problem”) to 5 (“a major problem”). The Cronbach’s alpha for this scale was calculated to be 0.88 in the current sample at the first administration of the measure.

#### Intrusive Thoughts

The Repetitive Thoughts Questionnaire (RTQ) (Feldman et al., 2010) asks participants to report on their experiences during a mindfulness exercise. The RTQ has a two-factor structure with 5 items assessing frequency of repetitive thoughts (e.g., thoughts about one or more problems in your life, a mental to-do list, criticisms of yourself) scored from 0 (never) to 4 (almost constantly). The second factor consists of 3 items assessing negative reactions to thoughts (e.g., to what degree were you upset, annoyed, or distracted by thoughts) scored from 0 (slightly or not at all) to 4 (extremely). Alpha reliability for frequency of repetitive thoughts was 0.83, and for negative reaction was 0.81 in a prior study (Johnson et al., 2014). In the current sample, the Cronbach’s alpha in the current sample was 0.76 for frequency, and 0.83 for negative reaction at baseline administration.

### Data Analysis

All analyses were conducted in SPSS Version 21 (IBM Corp, 2012). The study used a multilevel factorial design to test differential treatment effects with repeated measures over time. Variables values were screened for distribution assumptions prior to analysis.

#### Adherence

The rate of completion was calculated using the frequency (percentage) of cases in which the participant completed all four modules from the total number of cases. This calculation was performed for the aggregate sample, and for the supported and self-guided groups, independently. Between-group differences were assessed using a Chi-square analysis. A more nuanced examination of the rate and pattern of attrition was calculated by measuring the percent change (decrease) at each stage of the protocol. To better understand participants’ experience of obstacles and challenges to meditation and how these experiences may impact adherence, the total and mean scores for both administrations the OBS and RTQ were calculated for each of the study groups. A series of independent *t*-tests were used to examine the within-measure item differences between the first and second administrations of the OBS and RTQ. To better understand the impact of challenges and negatively appraised experiences on the participant’s progress through the intervention, regression analyses were performed using scores on the OBS and RTQ as the predictor variables. For these analyses adherence was treated as a continuous variable using the percentage of the intervention completed. The number and topic of communications between participants and study staff were also recorded and the frequency of participant-initiated communication for both the supported and self-guided groups analyzed for significant difference.

#### Benefits

Using a test-retest design, benefits obtained from completing the intervention were examined by conducting a series of paired *t*-tests (*p* = 0.05) calculating the mean differences in the baseline and final administration of the IBDQ, BAI, PSS, PHQ-9, MAAS, and MES for the aggregate sample. Cohen’s *d* (Cohen, 1992) was used to report effect size. To determine if group assignment affected benefits received from the intervention, mixed ANOVAs for each measure were conducted with group assignment (self-guided or supported) used as the between factor, and time (baseline and post-intervention) the within factor variable.

## Results

### Adherence

Out of the 108 participants who started the study (attempted baseline questionnaires), 33 participants completed the entire protocol, representing a completion rate of 30.6% for the aggregate sample. Of the 60 participants randomly assigned to the self-guided group, 7 completed the protocol, representing a completion rate of 11.7%. In the supported group, 26 of the 59 participants completed the entire study, representing a completion rate of 44.1%. No significant differences were observed on demographic or disease-related variables between the two groups (Table 3). Results of the Chi-square measuring the difference in completion rate between the supported and the self-guided groups indicated a significant difference (χ^2^ = 15.75; *p* = 0.000, *r* = 0.38) with an observed power of 0.976. A flow chart detailing drop-out points and the percent change at key points in the study design is provided in Figure 1.

**FIGURE 2:**
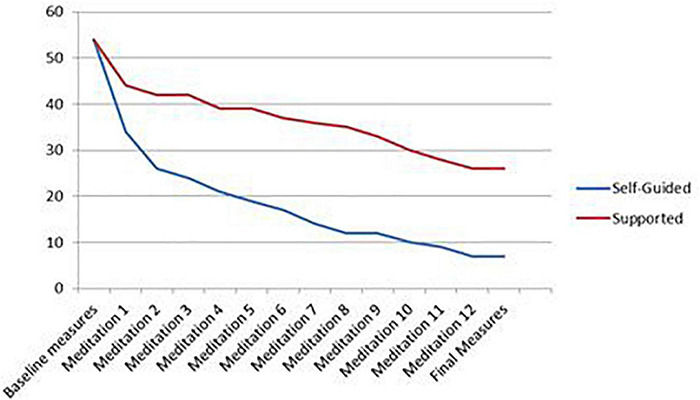
Participant drop-out over duration of study.

An examination of the points at which participants dropped out of the study for the aggregate sample revealed the largest decrease (−27.8%) occurred after the baseline measures were started, but before the first meditation was completed. In terms of participants who attempted at least one meditation, the largest decrease was found following the first meditation (23.1%). This pattern was found in both the self-guided and supported groups. A visual representation of drop-out data, by group, is found in Figure 2.

### Obstacles to Meditation

**FIGURE 3:**
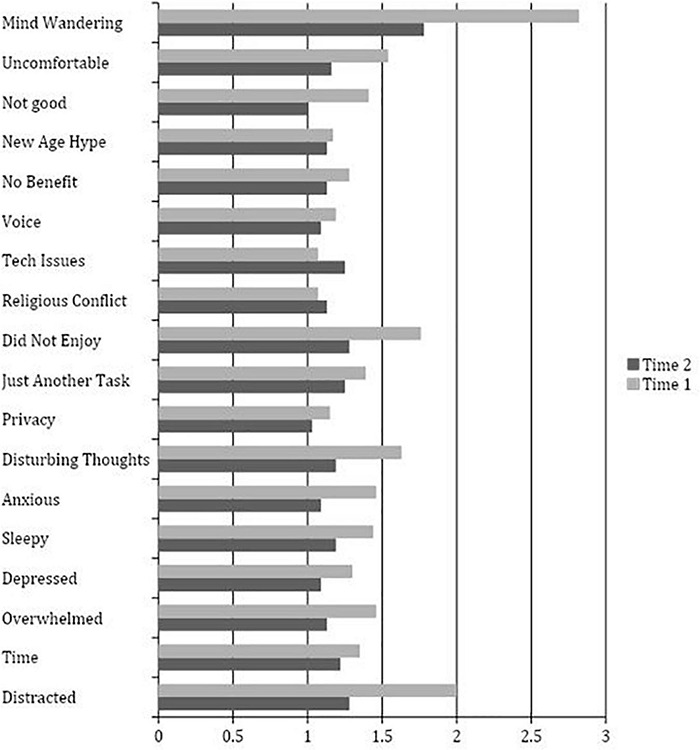
Obstacle Checklist Item Means at Time 1 (*n* = 78) and Time 2 (*n* = 33).

The mean scores of the OBS was 1.45 (SD = 0.47) and the RTQ was 12.86 (SD = 3.51) following the first meditation. Mean scores on these two measures did not significantly differ by study group (OBS: *p* = 0.30; RTQ: *p* = 0.64) or by study completion (OBS: *p* = 0.81; RTQ: *p* = 0.40). The change in the level of obstacles and repetitive thoughts experienced over the course of the intervention demonstrated no statistically different treatment effects, on either measure, between the self-guided and supported groups (OBS, *F* = 1.67, *p* = 0.205; RTQ, *F* = 0.101, *p* = 0.753). Results of the independent *t*-tests measuring test-retest differences in the first and second administrations of the OBS and RTQ are reported in Table 2. The three most intensely experienced obstacles endorsed on the OBS at the first administration were mind wandering (*M* = 2.82), feeling distracted (*M* = 2.00), and lack of enjoyment (*M* = 1.76). Mind wandering (*M* = 1.78), feeling distracted (*M* = 1.28), and lack of enjoyment (*M* = 1.28) were also the most experienced obstacles after the final mediation. Technical issues, an obstacle specific to online meditation instruction, was the only obstacle to increase from a mean of 1.07 at the first administration, to 1.25 at the final meditation. A bar graph of the full findings of this analysis is found in Figure 3.

**TABLE 2:** Change in challenges (*n* = 33).

	Time 1		Time 2			
Outcome	*M*	SD	*M*	SD	*t*	*p*
OBS	1.51	0.42	1.18	0.31	4.41	0.000**
RTQ	13.06	3.18	9.00	2.59	6.91	0.002**

**p < 0.01.

**FIGURE 4:**
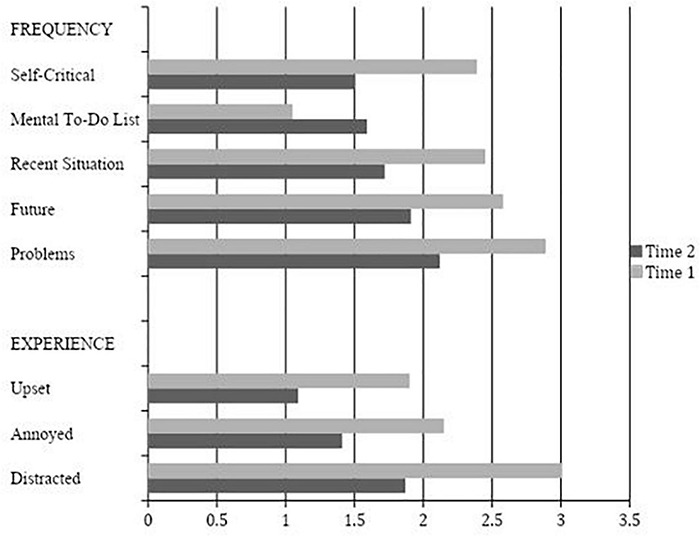
Repetitive Thoughts Questionnaire Item Means at Time 1 (*n* = 78) and Time 2 (*n* = 33).

Analysis of baseline RTQ items indicated the most frequent type of thought experienced was about problems in the participant’s life (*M* = 2.90), followed by worries about the future (*M* = 2.58), and recent situations the participant wished went differently (*M* = 2.45). Participants were more distracted by their thoughts (*M* = 3.01) than annoyed (*M* = 2.15) or upset (*M* = 1.90). The same pattern emerged in the final administration of the RTQ with thoughts regarding problems in the participant’s life (*M* = 2.12), followed by worries about the future (*M* = 1.91), and recent situations the participant wished went differently (*M* = 1.72) being the most frequently experienced items. Again, participants reported they were more distracted by their thoughts (*M* = 1.87) than annoyed (*M* = 1.41) or upset (*M* = 1.09). These findings are represented in Figure 4.

Results of the regression analysis measuring the effect obstacles and repetitive thoughts had on participant progress through the intervention were not significant for either the OBS (*p* = 0.07) or the RTQ (*p* = 0.49).

### Benefits

Benefits obtained from completing the online mindfulness intervention were measured by examining the difference in the scores on measures at baseline and following the intervention. Baseline correlation values for the measures included in the benefits analysis are shown in Table 3.

**TABLE 3:** Bivariate correlations between baseline measures (*n* = 108).

Variable	IBDQ	BAI	PSS	PHQ9	MASS
IBDQ					
BAI	0.52**				
PSS	0.64**	0.52**			
PHQ9	0.64**	0.65**	0.67**		
MASS	−0.12	−0.33**	−0.35**	−0.52*	
MES	−0.20*	−0.02	−0.18	−0.24*	0.40**

*p < 0.05; **p < 0.01.

Results of paired *t*-tests indicated that there was a significant change on the BAI, PSS, MAAS, and MES. No significant change was found in pre- and post-intervention administrations of the IBDQ or PHQ-9. Results of the analysis, including effect sizes, are summarized in Table 4.

**TABLE 4:** Change in outcome variables (*n* = 33).

	Time 1		Time 2				
Outcome	*M*	SD	*M*	SD	*t*	*p*	*d*
IBDQ	108.94	17.63	112.45	15.93	−1.868	0.072	0.21
BAI	12.12	8.49	7.27	6.35	4.14	0.000**	0.65
PSS	20.52	6.08	16.85	3.94	3.29	0.002**	0.72
PHQ-9	9.36	5.05	6.94	4.26	2.72	0.010*	0.52
MAAS	3.72	0.61	4.26	0.66	−4.49	0.000**	0.85
MES	76.64	8.97	88.18	7.13	−8.31	0.000**	1.42
OBS	1.51	0.42	1.18	0.31	4.41	0.000**	0.89
RTQ	13.06	3.18	9.00	2.59	6.91	0.002**	1.39

*p < 0.05; **p < 0.01.

Some of the largest changes were found on the MAAS and MES. The MES includes subscales measuring acceptance, awareness, non-reactivity, routine, distractibility, and unstructured patterns of relating to food and eating. An analysis of the MES subscales indicated significant increases on all subscales with the exception of distractibility. A summary of the results is found in Table 5. Results of mixed ANOVAs conducted for each measure demonstrated no statistically different treatment effects, on any measure, between the self-guided and supported groups (IBDQ, *F* = 0.06, *p* = 0.806; BAI, *F* = 2.92, *p* = 0.098; PSS, *F* = 0.15, *p* = 0.698; PHQ9, *F* = 0.50, *p* = 0.483; MAAS, *F* = 3.59, *p* = 0.068; MES, *F* = 0.89, *p* = 0.350).

**TABLE 5:** Change in MES subscales (*n* = 33).

	Time 1		Time 2				
Subscale	*M*	SD	*M*	SD	*t*	*p*	*d*
Acceptance	14.09	3.97	18.63	2.49	−6.75	0.000**	1.40
Awareness	14.34	2.67	16.06	2.34	−3.01	0.005**	0.64
Non-reactivity	13.19	2.37	14.78	1.53	−3.60	0.001**	0.78
Routine	13.41	2.39	13.63	1.62	−2.76	0.010*	0.11
Distractibility	12.06	2.09	12.72	1.85	−1.59	0.120	0.33
Unstructured	9.97	2.11	12.03	1.59	−6.14	0.000**	1.10

*p < 0.05; **p < 0.01.

## Discussion

Building on the growing body of literature reporting the benefits of mindful practice on health (Consedine and Butler, 2014; Greeson and Chin, 2019), the current study sought to better understand the feasibility and benefits of utilizing an online, mindfulness-based, intervention for people with IBD. This study also examined how different levels of support affected the participant experience. The intervention used was designed specifically to address psychosocial factors relevant to living with IBD. Modules were designed to address issues such as, body image, eating behaviors, coping with life’s difficulties, and self-compassion.

### Intervention Completion

People with IBD report barriers to traditional face-to-face interventions, including cost, physical limitations, and unpredictable symptoms that could be mitigated using online delivery. Despite the benefits an online delivery method may provide this population, the a rate of completion for the current study was only 30.6%, which is consistent with other online MBIs (36% for Ball et al., 2020; 52.3% for Cavanagh et al., 2014; 53.3% for Forbes et al., 2018; 23% for Howells et al., 2014; and 25% for Mikolasek et al., 2021). These studies cite loss of interest, health issues, and technical difficulties as factors inhibiting online intervention adherence.

The 30.6% completion rate was calculated using the aggregate sample. However, only 11.7% of participants in the self-guided group completed the interventions, as compared to 44% in the supported group. The large effect size for this finding indicates that levels of support influence participant retention. The results suggest that reminders, availability of a subject matter expert, and access to technical support may aid individuals in maintaining progress through an online MBI. This provides further evidence that including some level of support when administering interventions targeting health behaviors is associated with improved outcomes (Tate et al., 2006; Russell et al., 2018).

The optimal level of support may vary depending on the characteristics of the intervention used and the population targeted, and warrants further study. As the participant completed a module, the supported wing of the current study were sent automated, standardized, emails consisting of encouragement and an introduction of the material in the next module. Each email sent offered individualized support. Of the participants randomized to the supported group, 25.4%, utilized the bidirectional communication, emailing the researcher. The majority of these communications were regarding technical issues rather than the experience with the intervention.

### Benefits of the Intervention

Results of the current study indicate several psychological benefits following a MBI tailored to address the psychosocial correlates of IBD. A significant increase in dispositional mindfulness and mindful eating, as well as a significant decrease on measures of depression, stress, and anxiety were found. These findings add to the emerging body of research indicating benefits from brief online mindfulness training for people with chronic illnesses. Specific findings are further discussed in subsequent sections.

#### Level of Support

There was no difference in treatment effect, on any measure, between the self-guided and supported groups. The results suggest the intervention has benefits for those who complete it, regardless of the level of support received. This finding is consistent with previous research (Tate et al., 2006; Russell et al., 2018). Support appears to have a larger impact on completing the intervention rather than affecting the intervention’s benefits.

#### Mindfulness

A significant result in the current study was the increase in dispositional mindfulness scores. While perhaps unsurprising, the finding leads to the question of how increased mindfulness may benefit chronic illness populations. Research by Voth and Sirois (2009) found that self-blame and the utilization of avoidant coping strategies were associated with more difficulty adjusting to a diagnosis of IBD. Mindfulness training may benefit IBD patients by fostering disease acceptance and self-compassion rather than avoidance and self-blame.

A related mechanism by which mindfulness may benefit individuals with IBD is through moderating the effects of negative emotion. Individuals with IBD tend to score high on measures of neuroticism, a personality dimension closely related to the experience of anxiety and negative emotion (Sirois, 2015), and lower in psychosocial wellbeing as compared to healthy peers (Jordan et al., 2016). Research conducted by Wenzel et al. (2015) suggests mindfulness may serve as a buffer, moderating the effects of negative emotionality on mood and well-being. Neuroticism was more closely associated with depression in those measuring lower in levels of mindfulness. In the current sample, a reduction in distress related to intrusive thoughts was found. It is possible that while individuals high in neuroticism tend to experience negative thought patterns, applying the principles of mindfulness may reduce the impact of such patterns.

#### Stress and Anxiety

Results of the current study demonstrated statistically significant decreases on measures of perceived stress and anxiety following the intervention. This finding is especially important considering the strong evidence indicating deleterious effects of stress and anxiety on IBD symptoms and disease course (Dudley-Brown, 2002; Vidal et al., 2006; Wahed et al., 2010). Mindfulness may decrease stress and anxiety by encouraging processes of non-reactivity and acceptance (Didonna, 2009; Zou et al., 2020). Increased emotional control may lead to the utilization of more effective coping strategies, and in turn, reduce anxiety (Hill and Updegraff, 2012; Fraser, 2013; Garland and Roberts-Lewis, 2013). Mindfulness has also been associated with reduced anxiety through decreased rumination (Feldman et al., 2010; Raes and Williams, 2010; Burg and Michalak, 2011). Our findings from the RTQ demonstrated a reduction in the frequency and intensity of intrusive thoughts while meditating following the intervention. The practice of decentering one’s thoughts and feelings may have helped reduce anxiety in our sample.

#### Depression

The results of the current study are consistent with evidence indicating the efficacy of MBIs in reducing symptoms of depression (Hofmann et al., 2010; Kearney et al., 2012; Boggs et al., 2014). The current study extends the literature by demonstrating the efficacy of an online MBI for people with IBD, a population at risk for depression (Mittermaier et al., 2004; Graff et al., 2009). A systematic review found that people with comorbid anxiety and depression in IBD could benefit from non-pharmacologic interventions, yet often did not have access to interventions (Dubinsky et al., 2021). In a longitudinal study, Jordi et al. (2021) found that depression at enrollment was a robust risk factor for more severe IBD disease and inflammatory activity over time. Additionally, reduced depressive symptoms are associated with many health behaviors such as medication adherence, healthy diet, exercise, access to social support, and other behaviors that contribute to positive disease outcomes.

The mechanism by which mindfulness reduces depressive symptoms was outside the scope of the current study; however, Hofmann et al. (2010) posited that modifying the cognitive processes individuals utilize when experiencing negative emotion may reduce depressive symptoms. The mindfulness principle of non-judgmental awareness may allow for more neutral appraisal of typically negatively appraised stimuli. Depressive symptoms may decrease as one relates to thoughts and emotions with more neutrality.

#### Mindful Eating

The largest change was found in the increase on the MES. Many individuals with IBD engage in life-long refinement and modification of their diets in order to avoid GI distress (Gibson and Shepherd, 2010). These restrictive eating behaviors may place individuals with IBD at an increased risk for the development of eating disorders (Satherley et al., 2015), and research suggests a relationship between IBD and disordered eating (Ilzarbe et al., 2017). Several studies have demonstrated the efficacy of MBIs in reducing disordered eating behaviors, especially binging and craving-related eating (Kristeller et al., 2014; Fulwiler et al., 2015; Järvelä-Reijonen et al., 2018). The current study supports the theoretical basis for such interventions. Module 2 of the current study was devoted to providing mindful eating psychoeducation and guided meditations pertaining to awareness and acceptance of self-regulatory processes, including hunger and satiation. Analysis of the MES subscales indicated significant gains in the domains of acceptance, awareness, non-reactivity, routine, and structure. (The only subscale that did not demonstrate a significant change was distractibility). Mindfulness practice may reduce emotional reactivity, which can lead to eating to avoid difficult emotions and impulsive overeating (Forman and Butryn, 2015). Mindful eating is associated with better adherence to diet and reduced frequency of binge- and craving-related eating (Mason et al., 2018), all of which are associated with improved disease outcomes in IBD patients.

#### Quality of Life

Given the positive outcomes in terms of decreased depression, stress and anxiety, and increased dispositional mindfulness and mindful eating, it is somewhat surprising that quality of life, as measured by the IBDQ, did not significantly improve in the current sample. This trend is not without precedence in the literature (Jedel et al., 2014; Schoultz et al., 2015). Jedel et al. (2014) did not find a significant difference in quality of life, as measured by the IBDQ, following an 8-week MBSR intervention for UC patients. The researchers found that participants experiencing a flare during the intervention reported a decrease in quality of life. Similarly, Schoultz et al. (2015), applying a MBCT intervention to a sample of IBD patients, did not find a significant change in quality of life as measured by pre- and post-intervention administration of the IBDQ. The researchers offered several possible explanations including, an inadequate sample size (*n* = 22), questions regarding the instrument’s sensitivity, and extraneous factors not measured that could impact quality of life. The two studies described were both conducted face-to-face, but similar factors could apply to the current study.

The lack of change in the IBDQ, which measures symptom-specific quality of life, may be a function of the stages by which the quality of mindfulness is developed. The development of mindfulness begins with increased awareness and a “leaning in” to internal experience. For most able-bodied individuals, turning toward one’s internal bodily sensations is not met with as much pain and discomfort as for people living with IBD. The IBDQ may be measuring increased awareness of physical symptoms and the impact of those symptoms on the quality of life. Acceptance and non-judgment of difficult experiences, especially pain, take time. Our intervention may have been too brief to impact the IBDQ.

### Obstacles and Challenges to Meditation

The current study found that the level of obstacles and repetitive thoughts experienced after session one did not significantly predict how far a participant progressed through the intervention. However, our data suggest that participants who complete the intervention generally experienced fewer or less intense obstacles and intrusive thoughts while meditating at the end of the protocol than they did in the beginning. Average scores on both the OBS and the RTQ significantly decreased between the first and final meditations, similar to the experience of meditators in other studies (Fraser, 2013). Meditation may become easier with continued practice, even for individuals with chronic illnesses characterized by pain and discomfort. Despite an unchanged level of symptom-related distress, as measured by the IBDQ, participants reported a reduction in obstacles and a less negative appraisal of common challenges to meditation with continued practice. This finding is crucial because pain can increase negative appraisal of activities (Linton and Shaw, 2011), and a decrease in challenges reported may indicate an emerging ability to decenter from pain while meditating, which is consistent with current research in the field of brief MBIs for pain management (McClintock et al., 2019). Importantly, the change in these measures over the course of the intervention did not significantly differ based on group assignment, suggesting that the reduction in challenges is a function of meditation practice, rather than support.

### Limitations

#### Technical Considerations

The use of technology with consideration to recruitment, retention, and adherence is an ongoing challenge. In the current study, several participants in the supported group contacted researchers regarding difficulty logging onto the study website. While all technology issues were successfully addressed, it is unknown how frequently participant’s difficulties accessing the intervention went unreported or even led to dropout.

The current study utilized online recruitment, thus presuming a basic level of proficiency with technology. The assumption that people active in online forums would be comfortable accessing an online intervention, may not be accurate. Research suggests lack of computer savvy or access also may be a deterrent to using web-based therapies (Andersson and Titov, 2014; Ball et al., 2020). Though the future of managing chronic conditions, such as IBD, may incorporate online or smartphone interventions, this modality is not for everyone. For example, IBD is increasing in elderly populations (Bellone et al., 2021), who may have less comfort or exposure to technology highlighting the need for tailored interventions.

#### Meditation Difficulties

The information gained from the OBS and the RTQ measures is limited by the frequency of administration (following first and last meditation). Although the level of difficulty reported following the initial meditation did not predict how far a participant advanced through the protocol, we cannot assume difficulty while practicing meditations did not affect attrition. Future research designs that administer instruments measuring challenges and negative appraisal while meditating after every meditation (or specific intervals) may be better suited to understand the effect that frustration and expectation has on adherence to a meditation practice.

### Future Directions

In general, the results of the current study add to the literature evidencing the benefits and feasibility of online MBIs, and extend it to an IBD population. Additionally, results suggest that more support impacts retention and adherence. It should be noted that not all outcome measures demonstrated a significant benefit. Scores on the IBDQ did not show a significant change. If symptom-related changes take longer to manifest, then longitudinal research designs may better capture the effects of MBIs on symptom-specific quality of life. Future research which incorporates both longitudinal design and measures of both symptoms and psychological correlates of chronic illness (stress, anxiety, and depression) at intervals over the course of the study could provide valuable information regarding the pace of change. For example, because of the prominent role of psychological factors in chronic pain conditions such as chronic low back pain (Pincus and McCracken, 2013; Varallo et al., 2020, 2021) and fibromyalgia (Malin and Littlejohn, 2016; Varallo et al., 2021) future research should provide evidence for adherence and efficacy of mindfulness training also in these populations.

A whopping 97% of Americans own a smartphone (Pew Research Center, 2021). Research examining the feasibility and efficacy of using smartphone apps, rather than computer-based MBIs is still in a nascent stage. Smartphone apps may improve adherence because people tend to interact with their mobile devices frequently throughout the day. Additionally, mobile device applications allow the user access at their convenience or at crucial times when they need the intervention the most (Klasnja and Pratt, 2012). Research comparing a computer-based and smartphone-delivered intervention for weight loss found a higher retention (93% in the smartphone group versus 53% in the computer-based group) and improved outcomes for participants in the smartphone group (Carter et al., 2013). Additionally, a study focusing on the efficacy and feasibility of a smartphone-delivered mindful eating intervention found a significant reduction in craving-related eating (Mason et al., 2018). A logical next step in terms of research is to examine how smartphone-delivered MBIs may benefit individuals with IBD. The current study provided email reminders and technical assistance to the supported group, a function that could be built into a smartphone app. The benefit of bidirectional communication beyond that could be a focus of future research.

## Conclusion

Patients with IBD vary greatly in terms of psychological health, but in general, suffer high rates of depression and anxiety. Because the development and course of IBD may be significantly impacted by psychosocial factors, including chronic stress and anxiety, a holistic approach to treatment should aim to improve emotional health as well as physiological symptoms. The current study adds to the literature by demonstrating the benefits of a tailored online MBI for patients with IBD and examining the effects of different levels of intervention support. The intervention used was specifically designed to teach fundamentals of mindfulness while also addressing psychosocial and behavioral factors relevant to living with IBD. Psychotherapeutic interventions that promote disease acceptance and non-judgmental awareness may be especially well-suited for individuals with IBD, given the chronic nature of the disease. Mindfulness interventions may also effectively target behavioral factors, such as dysregulated eating, that impact disease progression and emotional health. While the evidence from the current and previous research supporting the efficacy of online MBIs is strong, attrition rates for such studies continues to be an issue. The current study demonstrates the benefit of bidirectional support over a self-guided experience in terms of adherence; however, even in the supported group only 44.1% completed the protocol. Future research may determine how to best utilize technology while still maintaining the integrity of the interventions and offering necessary support.

## Data Availability Statement

The raw data supporting the conclusions of this article will be made available by the authors, without undue reservation.

## Ethics Statement

The studies involving human participants were reviewed and approved by the University of North Carolina at Charlotte Internal Review Board (UNCC IRB). The patients/participants provided their written informed consent to participate in this study.

## Author Contributions

Both authors listed have made a substantial, direct, and intellectual contribution to the work, and approved it for publication.

## Conflict of Interest

The authors declare that the research was conducted in the absence of any commercial or financial relationships that could be construed as a potential conflict of interest.

## Publisher’s Note

All claims expressed in this article are solely those of the authors and do not necessarily represent those of their affiliated organizations, or those of the publisher, the editors and the reviewers. Any product that may be evaluated in this article, or claim that may be made by its manufacturer, is not guaranteed or endorsed by the publisher.
